# Neuroprotective effects of yoga practice: age-, experience-, and frequency-dependent plasticity

**DOI:** 10.3389/fnhum.2015.00281

**Published:** 2015-05-12

**Authors:** Chantal Villemure, Marta Čeko, Valerie A. Cotton, M. Catherine Bushnell

**Affiliations:** ^1^National Center for Complementary and Integrative Health, National Institutes of Health, Bethesda, MDUSA; ^2^Faculty of Dentistry, McGill University, Montreal, QCCanada; ^3^Integrated Program in Neuroscience, McGill University, Montreal, QCCanada; ^4^Department of Anesthesia, McGill University, Montreal, QCCanada

**Keywords:** yoga, age-related gray matter decline, neuroprotection, magnetic resonance imaging, voxel-based morphometry

## Abstract

Yoga combines postures, breathing, and meditation. Despite reported health benefits, yoga’s effects on the brain have received little study. We used magnetic resonance imaging to compare age-related gray matter (GM) decline in yogis and controls. We also examined the effect of increasing yoga experience and weekly practice on GM volume and assessed which aspects of weekly practice contributed most to brain size. Controls displayed the well documented age-related global brain GM decline while yogis did not, suggesting that yoga contributes to protect the brain against age-related decline. Years of yoga experience correlated mostly with GM volume differences in the left hemisphere (insula, frontal operculum, and orbitofrontal cortex) suggesting that yoga tunes the brain toward a parasympatically driven mode and positive states. The number of hours of weekly practice correlated with GM volume in the primary somatosensory cortex/superior parietal lobule (S1/SPL), precuneus/posterior cingulate cortex (PCC), hippocampus, and primary visual cortex (V1). Commonality analyses indicated that the combination of postures and meditation contributed the most to the size of the hippocampus, precuneus/PCC, and S1/SPL while the combination of meditation and breathing exercises contributed the most to V1 volume. Yoga’s potential neuroprotective effects may provide a neural basis for some of its beneficial effects.

## Introduction

Yoga originates in India and is increasingly practiced by Westerners (Barnes et al., 2004, 2008; Saper et al., 2004; Birdee et al., 2008). Several hatha yoga styles are practiced in western societies and most of them encompass physical postures (termed *asana* in Sanskrit), breath control exercises (*pranayama*), and meditation (*dhyana*) including the chanting of Sanskrit mantras.

Yoga offers several documented health benefits including, but not limited to, improvement of depressive, anxious and stressful states and the relief of various painful conditions (Woolery et al., 2004; Lavey et al., 2005; Shapiro et al., 2007; Wren et al., 2011; Li and Goldsmith, 2012). However, the effects of long-term regular yoga practice on the central nervous system had not been explored until recently when it was shown that experienced yoga practitioners have greater GM volume than matched controls in several brain regions including the hippocampus, primary and secondary somatosensory cortices (S1 and S2), insular cortex, anterior, and posterior cingulate cortices (ACC and PCC), inferior and superior parietal cortices, superior temporal gyrus, orbitofrontal cortex (OFC), medial prefrontal cortex, and cerebellum (Froeliger et al., 2012; Villemure et al., 2013). Nevertheless, the cross-sectional nature of these studies does not permit attributing these group differences to yoga practice with certainty, since people with a given brain structure might, for some reason, be drawn to practice yoga.

In the current report, we revisit our data set to address whether the number of years of yoga experience, the amount of weekly yoga practice, and the different aspects of yoga practice impact specific brain regions. Brain differences related to experience and amount of practice within a group of yoga practitioners would suggest that yoga contributes to changing brain anatomy. Indeed, both short-term and long-term increased training and/or performance have been associated with GM increases in human adults in a wide range of cognitive tasks (Maguire et al., 2000; Golestani et al., 2002; Mechelli et al., 2004; Lazar et al., 2005; Draganski et al., 2006; Holzel et al., 2008; Grant et al., 2010) and motor skills (Sluming et al., 2002; Draganski et al., 2004; Driemeyer et al., 2008) in the brain areas involved in those tasks.

Additionally, if different aspects of yoga practice such as postures, breath control techniques, and meditation contributed differently to brain changes it would further suggest that yoga practice contributes to changing brain anatomy. For example, meditation and physical activity are associated with structural differences in brain regions that do not completely overlap. Meditators were repeatedly shown to have larger hippocampal (Holzel et al., 2008; Luders et al., 2009, 2013a,b), insular (Lazar et al., 2005; Holzel et al., 2008; Luders et al., 2012), and left inferior temporal gyrus volume than controls (Holzel et al., 2008; Luders et al., 2009; Leung et al., 2013), while a recent review of the literature revealed that greater cardiorespiratory fitness and physical activity were most consistently associated with larger hippocampal and prefrontal GM volume (Erickson et al., 2014). Given that hatha yoga is a meditative practice embodied in physical postures, it is likely that we could uncover brain areas whose GM is more likely influenced by either postures, breath control, meditation, or different combinations of these.

Finally, previous studies have shown that global brain GM declines with age (Good et al., 2001; Salat et al., 2004; Ziegler et al., 2012) while physical activity and cardiovascular fitness (Colcombe et al., 2003; Tseng et al., 2013), as well as meditation (Lazar et al., 2005; Pagnoni and Cekic, 2007; Luders et al., 2015) have been associated with age-related neuro-protection. In the current report we evaluate whether yoga practice also offers a global age-related protective effects on brain GM volume, since yoga encompasses both a physical and a meditative component. Together, such findings would strongly suggest that yoga practice impacts the brain rather than yoga practitioners having fundamentally larger brain volumes in certain areas leading them to adopt yoga.

## Materials and Methods

### Participants

**FIGURE 1:**
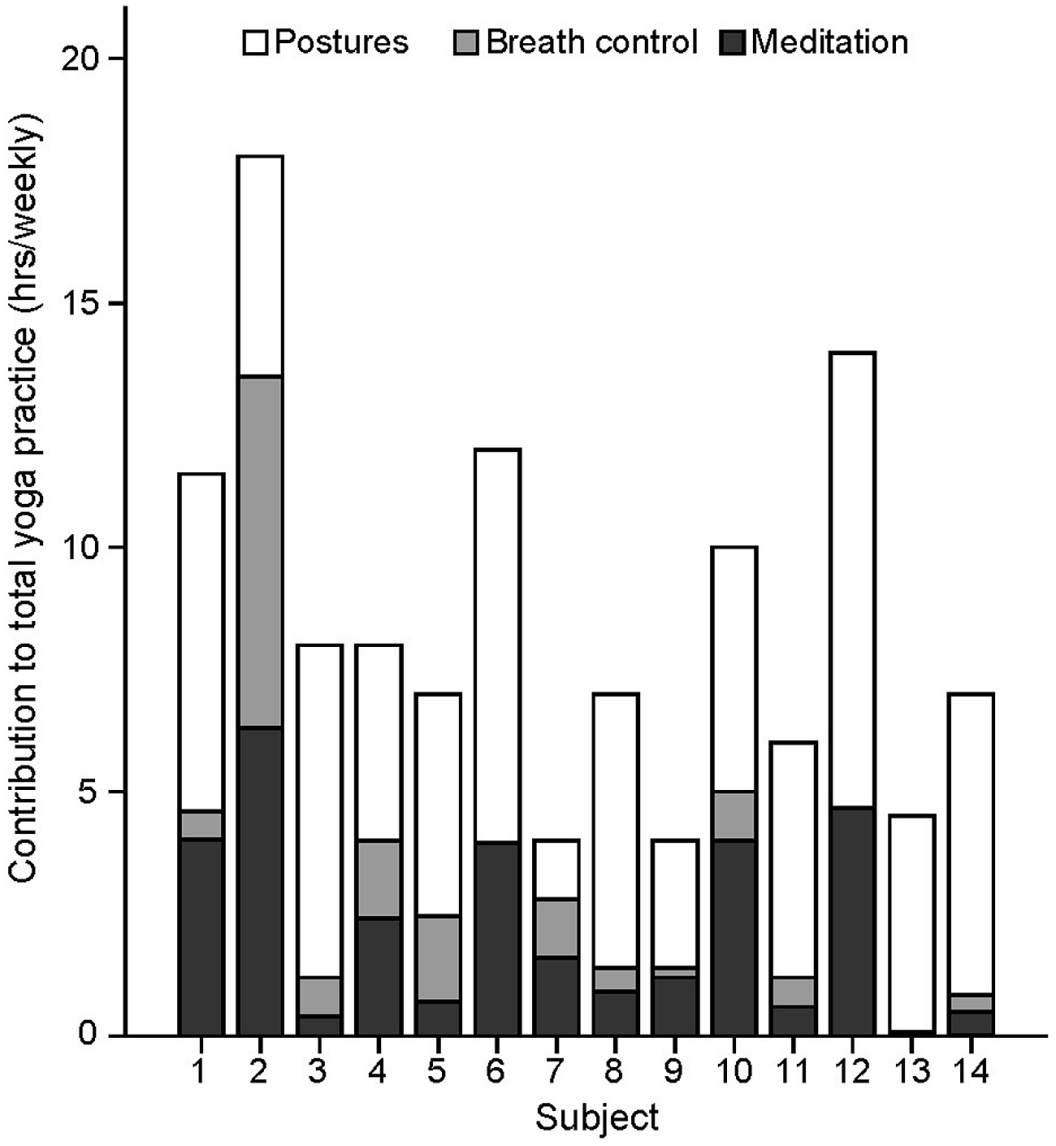
**Repartition of yoga practice into its three main components**. Number of hours devoted weekly to yoga postures, yoga-related meditation, and separate breath control exercises (excluding breath awareness during postures) for each yoga practitioner. Only the time devoted to the practitioners’s personal practice (excluding teaching) are reported.

This study was approved by McGill University Institutional Review Board. Data were drawn from the same study population described in Villemure et al. (2013). We recruited 14 experienced yoga practitioners from ads posted at yoga studios in Montreal, Canada. The study was open to all types of yoga that integrated physical postures, breath control exercises (minimally breath awareness during postures), and concentration/meditation practices including chanting Sanskrit mantras. During a telephone screening we asked yoga practitioners several questions about their yoga practice and experience. Subjects were questioned about the number of years they had been practicing yoga (experience) and asked to evaluate separately how many hours/week they devoted to their current personal yoga practice and to the teaching of yoga if they were yoga teachers. Subjects were then asked to report what proportion of their current personal weekly yoga practice (excluding teaching) consisted of these three subcomponents: postures, stand-alone breathing exercises, and yoga-related meditation including chanting (**Figure 1**). Fourteen physically active controls were recruited from ads posted on McGill’s online classifieds and were individually matched to yogis in terms of sex, age, body mass index, handedness, education, and exercise level outside of yoga (**Table 1**). Applicants were excluded if they currently suffered from or had a history of claustrophobia, chronic pain, chronic systemic diseases, psychiatric, or neurological disorders, or if they were pregnant or breast-feeding, regular smokers or regular users of marijuana, alcohol, or any other recreational drugs, or taking analgesics or anti-depressants. Controls were excluded if they had any previous experience with yoga, meditation or martial arts. All participants provided written informed consent and received monetary compensation for their participation.

**Table 1:** Group matching criteria and yoga practice characteristics.

	Yogis (*N* = 14)	Controls (*N* = 14)	
Sex	Five males Nine females	Five males Nine females	
Handedness	Nine right-handed Five left handed	Nine right-handed Five left handed	
Age (years)	37.0 ± 6.6	36.7 ± 7.3	*t*(df = 26) = 0.11; *p* = 0.913
Body mass index	21.6 ± 2.1	22.6 ± 2.8	*t*(df = 26) = 1.12; *p* = 0.271
Education (years)	15.9 ± 1.6	15.5 ± 2.1	*t*(df = 26) = 0.61; *p* = 0.548
Exercise (h/week)	5.2 ± 3.1	4.7 ± 3.5	*t*(df = 26) = 0.41; *p* = 0.684
Yoga experience (years)	9.6 ± 2.8 Range: 6–16		
Weekly yoga practice (h/week)	8.6 ± 4.1 Range: 4–18		
Physical postures (%) Concentration and meditation (%) Breath control exercises (%)	66 ± 21 23 ± 14 11 ± 12		
Yoga teachers (N) Teaching (h/week)	11 9.1 ± 5.1 Range: 3–17.5		

*Values are means ± SD.*

### MRI Acquisition

Subjects participated in one MRI scanning session including a 10-min anatomical scan and a 15-min diffusion tensor imaging (DTI) scan (DTI results are reported elsewhere; Villemure et al., 2013). Participants wore earplugs to protect them from the scanner noise and their heads were immobilized. Brain images were acquired using a 3 Tesla Siemens Trio (Siemens, Erlangen, Germany) with a standard 12-channel head coil. T1-weighted images were acquired for each subject using a 3D MP-RAGE (Magnetization Prepared Rapid Acquisition by Gradient Echo) sequence [inversion time (TI) = 900 ms, repetition time (TR) = 2300 ms, echo time (TE) = 2.98 ms, flip angle = 9°, filed of view (FOV) = 256 mm; voxel size 1 mm^3^).

### Voxel-Based Morphometry (VBM)

#### Preprocessing

Anatomical images were preprocessed with the VBM8 toolbox^1^ for SPM8^2^, running on Matlab (R2007a, The Mathworks, Natick, MA, USA). Details are reported in Villemure et al. (2013) but briefly, images were first bias corrected, tissue classified, and spatially normalized to the MNI space to allow comparison of voxel-based morphometry (VBM) results across studies. The voxel values were multiplied by the non-linear components derived from the spatial normalization to allow for the comparison of the absolute amount of GM volume corrected for individual brain sizes. The modulated volumes were smoothed with a Gaussian kernel of 8 mm full width at half maximum (FWHM).

#### Correlating Total GM Volume with Age in Yogis and Controls

Total GM volume [including the cerebellum and expressed as % of total intracranial volume (TIV)] was correlated with age separately for each group using Pearson correlations in SPSS PASW Statistics 18.0.

#### Effects of Years of Yoga Experience and Hours of Weekly Yoga Practice on Brain GM Volume

For the yoga practitioners, we performed two separate whole-brain VBM regression analyses while controling for age and using the number of years of yoga experience and the weekly amount (hours) of yoga practice as predictors [voxel-wise threshold *p* < 0.001, cluster corrected at *p*< 0.05 using random-field-theory (RFT)].

#### Examining Which Aspects of Yoga Practice Best Predict GM Volumes of the Brain Areas that Correlate with Weekly Amount of Yoga Practice

Once the significant clusters related to the number of hours of weekly yoga practice were identified in the whole-brain regression analyses, we extracted their volumes (in arbitrary units) for each yoga practitioner using MarsBar toolbox for SPM and used the number of weekly hours devoted to the practice of postures, breath control, and yoga-related meditation to determine which aspect or combination of aspects of the total personal yoga practice (excluding teaching) best predicted the size of the identified brain regions using standard multiple regression analyses (SPSS PASW Statistics 20.0) and commonality analyses (SAS 9.3). Regression commonality analysis enables the partitioning of the *R*^2^ effect sizes into the effects uniquely explained by each predictor and the effects commonly explained by all possible combination of predictors (Seibold and McPhee, 1979; Reichwein Zientek and Thompson, 2006). Such analyses were not possible for the number of years of yoga practice for lack of a good estimate of life-time practice in the three different spheres of practice (postures, breath control, and meditation).

### Additional Correlation Analysis

The number of years of yoga experience was correlated with the number of hours spent teaching yoga each week using Pearson correlations in SPSS PASW Statistics 18.0.

## Results

### Long-Term Yoga Practice May Have Prevented the Typically Observed Age-Related Decline of GM

**FIGURE 2:**
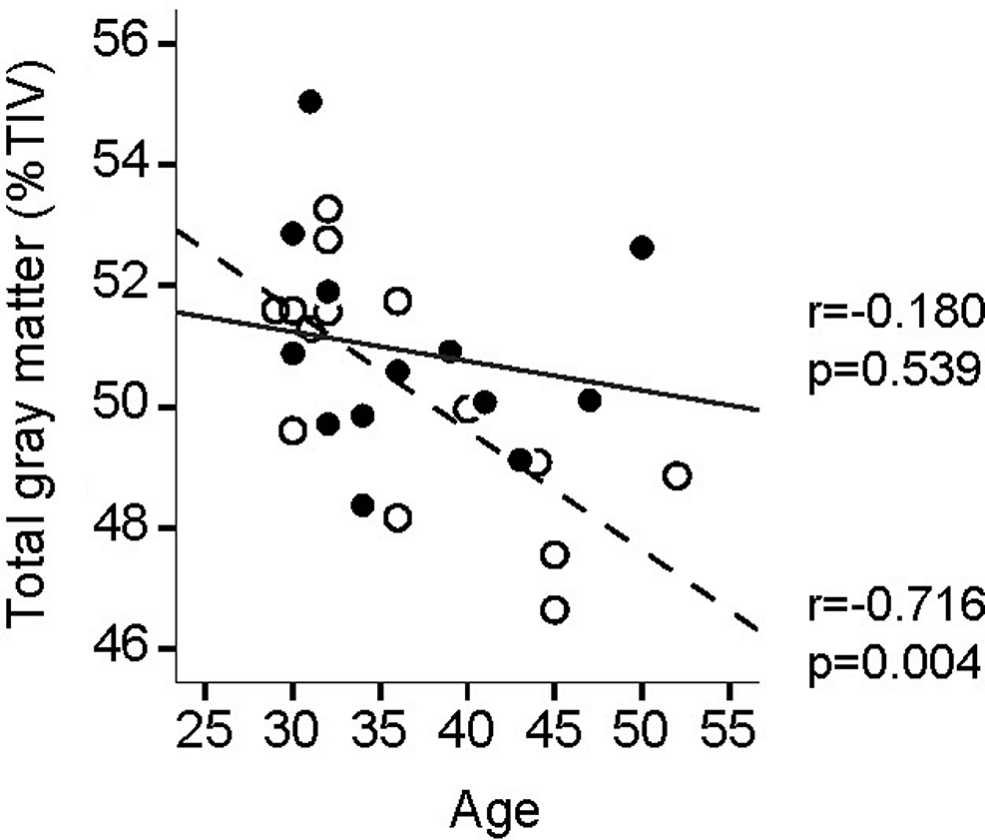
**Age-related whole brain gray matter (GM) volume decline**. Full circles and full fit line, yogis; open circles and dotted fit line, controls.

In controls, whole brain GM negatively correlated with age [GM volume (**Figure 2**): *r* = –0.716, *p* = 0.004]. In yogis there was no such correlation [GM volume (**Figure 2**): *r* = –0.18, *p* = 0.539]. However, the differences in slopes did not reach statistical significance [group x age interaction: *F*(1,24) = 2.555, *p* = 0.123].

### GM Volumes of Several Brain Regions Are Related to the Number of Years of Experience and the Number of Hours of Weekly Yoga Practice

**FIGURE 3:**
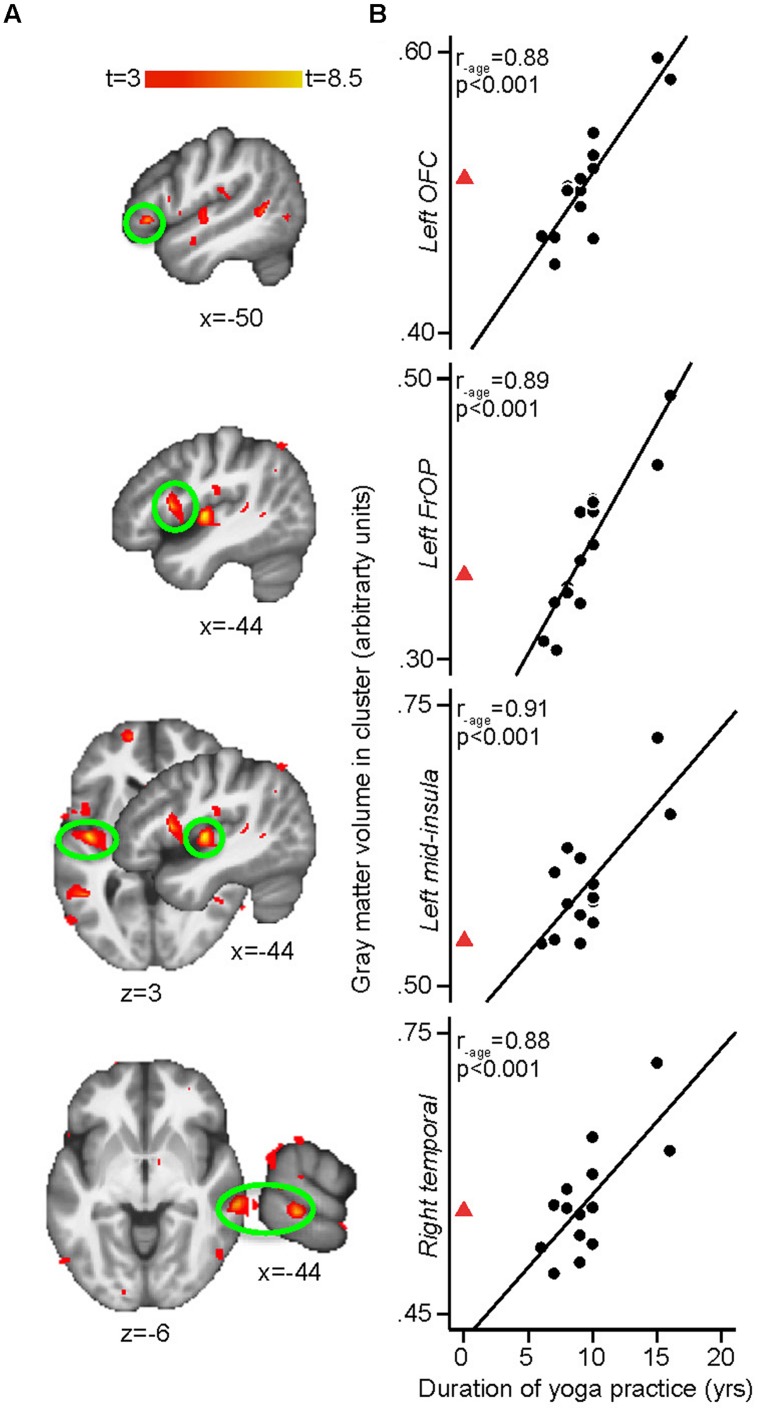
**(A)** Whole-brain voxel-based morphometry (VBM) regressions with duration (in years) of yoga practice controlling for age. Duration of yoga practice is associated with more GM in the left insula [peak *x, y, z* MNI coordinates: –44, –12, 3; *t*(peak) = 8.38; cluster size: 1374 mm^3^, *p*(cluster) = 0.002], left frontal operculum (BA 44) [–44, 11, 10; *t*(peak) = 7.57; cluster size: 263 mm^3^, *p*(cluster) = 0.006], right middle temporal gyrus (BA 21) [64, –31, –6; *t*(peak) = 7.00; cluster size: 557 mm^3^, *p*(cluster) = 0.011], and left orbitofrontal cortex (OFC; BA 47) [–50, 27, –3; *t*(peak) = 6.53; cluster size: 165 mm^3^, *p*(cluster) = 0.018]. Results are cluster-corrected at *p* < 0.05. **(B)** Scatterplots show the extracted mean GM values in the significant clusters plotted against years of yoga practice for each yoga practitioner (arbitrary units, a.u.). Full black circles illustrate individual yoga practitioners and fit line shows regression line across the yoga group. Red triangle shows the mean of control values extracted from the same GM cluster (i.e., representing zero year of yoga practice). The red triangle is not included in the regression with GM, but shows the natural extension at 0 year of yoga practice (extrapolated from control subjects) and is displayed here demonstrate that no yoga experience and the most yoga experience fall onto the opposite end of the same spectrum. Displayed *R*-values are derived from correlating the mean GM in each significant cluster with yoga duration, while controlling for age (SPSS, two-tailed *p*-values).

**FIGURE 4:**
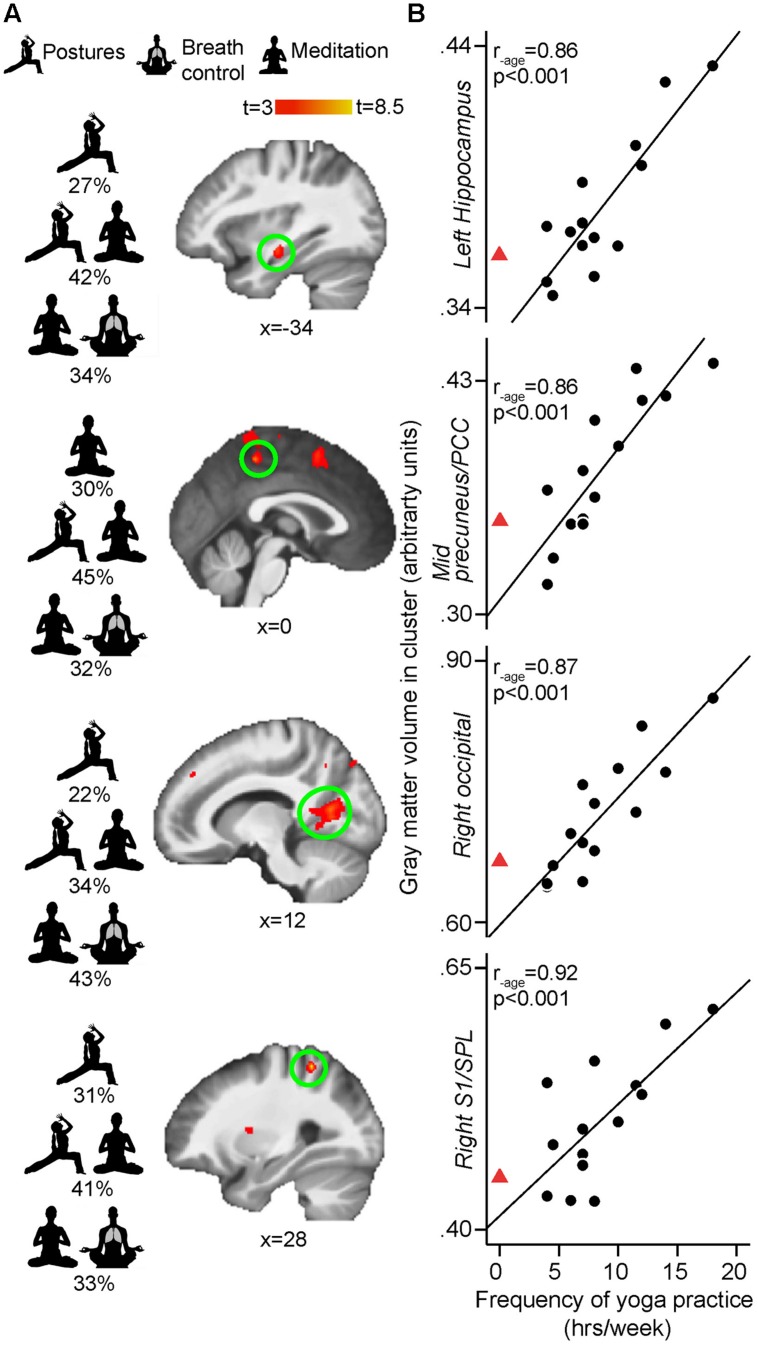
**(A)** Whole-brain VBM regressions with frequency (hours per week) of yoga practice controlling for age. Increased frequency of yoga practice is associated with more GM in right S1/SPL (BA 7/5) [peak *x, y, z* MNI coordinates: 28, –45, 57; *t*(peak) = 8.17; cluster size: 172 mm^3,^, *p*(cluster) = 0.005], left hippocampus [–34, –16, –14; *t*(peak) = 6.20; cluster size: 138 mm^3^, *p*(cluster) = 0.02], mid precuneus/PCC (BA 7/31) [0, –37, 52; *t*(peak) = 6.15; cluster size: 88 mm^3^, *p*(cluster) = 0.033], and right occipital cortex (BA 17) [12, –63, 12; *t*(peak) = 5.79; cluster size: 499 mm^3^, *p*(cluster) = 0.013]. Results are cluster-corrected at *p* < 0.05 and displayed on study average brain (*n* = 28). SPL, superior parietal lobule; PCC, posterior cingulate cortex. The main aspects or combination of aspects of yoga practice contributing to each GM volume size according to the regression commonality analyses are depicted to the left of the brains. **(B)** Scatterplots show the extracted mean GM values in the significant clusters plotted against frequency of yoga practice for each yoga practitioner (arbitrary units, a.u.). Full black circles illustrate individual yoga practitioners and fit line shows regression line across the yoga group. Red triangle shows the mean of control values extracted from the same GM cluster (i.e., representing zero weekly hour of yoga practice). The red triangle is not included in the regression with GM, but shows the natural extension at 0 hour/week of yoga practice (extrapolated from control subjects) and is displayed here to demonstrate that no yoga practice and the most yoga practice falls onto the opposite end of the same spectrum. Displayed *R*-values are derived from correlating the mean GM in each significant cluster with yoga frequency, while controlling for age (SPSS, two-tailed *p*-values)

The whole brain regression analyses conducted in yogis revealed that the number of years of yoga experience was positively correlated with GM volume in clusters located in the left mid-insula, left frontal operculum (Brodmann area [BA] 44), right middle temporal gyrus (BA 21) and left OFC (BA 47; **Figure 3**). The current weekly amount of yoga practice was positively correlated with GM volume in clusters located in the right primary somatosensory cortex/superior parietal lobule (S1/SPL), left hippocampus, midline precuneus/PCC, and right primary visual cortex [V1, (BA 17)] (**Figure 4**).

### Subcomponents of Weekly Yoga Practice Differentially Predict GM Volumes in the Brain Areas Found to Correlate with Total Weekly Yoga Practice

The number of hours devoted weekly to yoga postures, yoga-related meditation, and stand-alone breath control exercises for each yogi are presented in **Figure 1** and were used in separate standard multiple regression analyses to determine which factor or combination of factors best predicted the volumes of the left hippocampus, right S1/SPL, right V1, and midline precuneus/PCC. The correlations between variables are shown in **Table 2**. Multicollinearity was minimal here since there were no significant correlations between predictor variables except for a tendency toward a positive correlation between the number of hours devoted to meditation and breath control exercises. The prediction models for all four regions using all three predictors were statistically significant [hippocampus: *F*(3,10) = 8.848, *p* = 0.004; S1/SPL: *F*(3,10) = 3.948, *p* = 0.043; V1: *F*(3,10) = 10.631, *p* = 0.002; precuneus/PCC: *F*(3,10) = 14.529, *p* = 0.001] indicating that the prediction of these four brain GM volumes using these three aspects of yoga practice was accomplished better than can be expected by chance alone. These results remained significant when using a criterion *p* adjusted for the number of regression analyses performed (*p*< 0.0125) except for S1/SPL. Detailed multiple linear results are shown in **Table 3**.

**Table 2:** Correlation matrix associated with the multiple regression analysis.

	Postures	Yoga-related meditation	Stand-alone breath control exercises
Postures		*r* = 0.332, *p* = 0.246	*r* = –0.246, *p* = 0.396
Yoga-related meditation			*r* = 0.525, *p* = 0.054
S1/SPL	*r* = 0.523, *p* = 0.054	***r* = 0.611, *p* = 0.020**	*r* = 0.337, *p* = 0.238
V1	*r* = 0.506, *p* = 0.064	***r* = 0.754, *p* = 0.002**	*r* = 0.525, *p* = 0.054
Hippocampus	***r* = 0.591, *p* = 0.026**	***r* = 0.729, *p* = 0.004**	*r* = 0.393, *p* = 0.164
Precuneus/PCC	***r* = 0.554, *p* = 0.040**	***r* = 0.854, *p* = 0.000**	*r* = 0.358, *p* = 0.208

*Pearson correlations between the different independent variables (predictors) as well as between the independent and dependent variables. Listed *p-*values are two-tailed. Significant results are bolded. S1, primary somatosensory cortex; SPL, superior parietal volume; V1, primary visual cortex; PCC, posterior cingulate cortex.*

**Table 3:** Multiple regression results.

	*R*	*R*^2^	Adjusted *R*^2^	*B*	Beta	*p*	*r*	*r*^2^	*r*_sp_	*r*_sp_^2^	*r*_struc_	*r*_struc_^2^	PM
**Hippocampus**
Postures	0.852	0.726	0.644	0.007	0.549	0.024	0.591	0.349	0.441	0.194	0.694	0.481	0.324
Meditation	0.852	0.726	0.644	0.005	0.372	0.144	0.729	0.531	0.262	0.069	0.856	0.732	0.271
Breath	0.852	0.726	0.644	0.005	0.333	0.175	0.393	0.154	0.241	0.058	0.461	0.213	0.131
**S1/SPL**
Postures	0.736	0.542	0.405	0.015	0.510	0.084	0.523	0.274	0.410	0.168	0.711	0.505	0.267
Meditation	0.736	0.542	0.405	0.008	0.275	0.386	0.611	0.373	0.194	0.038	0.830	0.689	0.168
Breath	0.736	0.542	0.405	0.010	0.318	0.307	0.337	0.114	0.230	0.053	0.458	0.210	0.107
**V1**
Postures	0.873	0.761	0.690	0.017	0.512	0.024	0.506	0.256	0.411	0.169	0.580	0.336	0.259
Meditation	0.873	0.761	0.690	0.012	0.335	0.157	0.754	0.569	0.236	0.056	0.864	0.746	0.253
Breath	0.873	0.761	0.690	0.018	0.475	0.050	0.525	0.276	0.344	0.118	0.601	0.362	0.249
**Precuneus/PCC**
Postures	0.902	0.813	0.757	0.006	0.337	0.076	0.554	0.307	0.271	0.073	0.614	0.377	0.187
Meditation	0.902	0.813	0.757	0.013	0.704	0.005	0.854	0.729	0.497	0.247	0.947	0.896	0.601
Breath	0.902	0.813	0.757	0.001	0.071	0.713	0.358	0.128	0.052	0.003	0.397	0.158	0.025

*R, raw regression coefficient; *B*, raw regression coefficient; Beta, standardized regression coefficient; *p*, probability; *r*, zero-order correlation (Pearson correlation); *r_*sp*_*, semipartial correlation; *r*_*sp*_^2^, commonality coefficient for the unique effects; *r_*struc*_*, structure coefficient (*r*/*R*); PM, product measure (*r* × Beta). S1, primary somatosensory cortex; SPL, superior parietal volume; V1, primary visual cortex; PCC, posterior cingulate cortex.*

#### Predicting Hippocampal Volume

The model including the three predictors accounted for approximately 73% of the hippocampal volume variance. The *Beta* coefficient with large structure coefficients indicated that the number of hours devoted weekly to the practice of postures was a good predictor of hippocampal GM volume (Reichwein Zientek and Thompson, 2006). Product measures enable rank ordering of variable importance based on the partitioning of the regression effect (Nathans et al., 2012). These values indicated that the practice of postures followed by yoga-related meditation were the best predictors of hippocampal GM volume. This was confirmed by the commonality analysis results (**Table 4**) showing that nearly half (42%) of the variance in hippocampal GM volume explained by the predictors was common to postures and meditation.

**Table 4:** Commonality matrix.

	Coefficient	% TOTAL
**Hippocampus**
Unique to postures (x1)	0.194	26.722
Unique to meditation (x2)	0.069	9.504
Unique to breath control (x3)	0.058	7.989
Common to x1, x2	0.308	42.424
Common to x1, x3	-0.058	-7.989
Common to x2, x3	0.250	34.435
Common to x1, x2, x3	-0.095	-13.085
Total	0.726	100
**S1/SPL**
Unique to postures (x1)	0.168	30.996
Unique to meditation (x2)	0.038	7.011
Unique to breath control (x3)	0.053	9.779
Common to x1, x2	0.223	41.144
Common to x1, x3	-0.053	-9.779
Common to x2, x3	0.177	32.657
Common to x1, x2, x3	-0.064	-11.808
Total	0.542	100
**V1**
Unique to postures (x1)	0.169	22.208
Unique to meditation (x2)	0.056	7.359
Unique to breath control (x3)	0.118	15.506
Common to x1, x2	0.260	34.166
Common to x1, x3	-0.095	-12.484
Common to x2, x3	0.331	43.495
Common to x1, x2, x3	-0.078	-10.250
Total	0.761	100
**Precuneus/PCC**
Unique to postures (x1)	0.073	8.979
Unique to meditation (x2)	0.246	30.258
Unique to breath control (x3)	0.002	0.246
Common to x1, x2	0.366	45.018
Common to x1, x3	0.009	1.107
Common to x2, x3	0.259	31.857
Common to x1, x2, x3	-0.142	-17.466
Total	0.813	100

*S1, primary somatosensory cortex; SPL, superior parietal volume; V1, primary visual cortex; PCC, posterior cingulate cortex.*

#### Predicting S1/SPL Volume

The model including the three predictors accounted for approximately 54% of the volume variance of S1/SPL with the number of hours devoted to postures having the greatest influence (highest *Beta* with a large structure coefficient). This was confirmed by the product measure value and the commonality analysis showing that 31% of the variance explained by the predictors was explained by postures alone while 41% was explained by the combination of postures and meditation.

#### Predicting V1 Volume

The model including the three predictors accounted for about 76% of V1 volume variance. The *Beta* coefficients accompanied by large structure coefficients indicated that both postures and breath control exercises were good predictors of V1 volume. However, the product measure coefficients revealed that all three measured dimensions of yoga practice almost equally contributed. The commonality analysis showed that these factors worked best in combination with nearly half (44%) of the explained variance of V1 volume being explained by the combination of meditation and breath control exercises.

#### Predicting Precuneus/PCC Volume

The model including the three predictors accounted for approximately 81% of the volume variance of precuneus/PCC. Yoga-related meditation was by far the best predictor of GM precuneus/PCC volume (high beta coefficient, large structure coefficient, and product measure value). This was confirmed by the commonality analysis revealing that nearly a third of the explained variance of the precuneus/PCC GM volume was explained by meditation alone, and 45% by the combination of meditation and postures.

In summary, the combination of postures and meditation contributed the most to the size of the hippocampus, precuneus/PCC, and S1/SPL while the combination of meditation and breath control exercises contributed the most to V1 size (**Figure 3**).

### The More Experienced Yogis Spend More Time Teaching Yoga Each Week

If we exclude an outlier who had the least amount of yoga experience but was teaching many hours/week and one teacher for whom we had no data about the number of hours of weekly teaching, we find a positive correlation between the number of years of yoga experience and the number of hours spent teaching yoga each week (*R* = 0.679; *p* = 0.015, 2-tailed; *N* = 12).

## Discussion

These data suggest that yoga practice has a neuroprotective effect against the well-documented age-related whole-brain GM degradation, which was evident in our control group. The data also revealed that increasing experience (years of yoga practice) had a differential effect on the brain than did increasing weekly hours of yoga practice. Whole-brain regression analyses showed that more years of yoga experience was associated with increasing GM volumes in clusters located in the left insula, left frontal operculum, right middle temporal gyrus, and left OFC, while more hours devoted to yoga weekly was associated with increasing GM volumes in the right S1/SPL, left hippocampus, midline precuneus/PCC, and right V1 cortex. Finally, postures, breathing exercises, and meditation contributed differently to the structural changes of the four brain areas associated with the amount of weekly yoga practice, consistent with the different nature of the processing taking place in those structures.

### Age-Related Global GM Matter Decline

Global brain GM declines with age (Good et al., 2001; Salat et al., 2004), and we found this same decline in our physically active healthy controls but not in the yoga group suggesting that yoga practice may offer a neuroprotective effect against age-related GM loss. However, because the differences in age-related GM decline slopes did not reach statistical significance, possibly due to a lack of power ensuing from our relatively small sample size, this finding should be interpreted with caution. Physical activity and cardiovascular fitness (Colcombe et al., 2003; Tseng et al., 2013), as well as meditation (Lazar et al., 2005; Pagnoni and Cekic, 2007; Luders et al., 2015) offer similar neuroprotective advantages, suggesting that both the physical and meditative aspects of yoga may contribute to the protection against age-related decline in GM. The differential influence of postures and meditative practices on the brain, as shown here, support that both physical and mental activities contribute to total volume changes, but may occur in partially separate regions. Whether yoga, which encompasses both a physical and a meditative aspect, offers increased protection over meditation or physical activity alone remains an open question.

### GM Volume Related to Yoga Experience

More GM related to long-term experience or skill proficiency have been reported in a number of populations like meditators (Lazar et al., 2005; Holzel et al., 2008; Grant et al., 2010; Luders et al., 2013a), orchestra musicians (Sluming et al., 2002), taxi drivers with extensive navigation experience (Maguire et al., 2000), and bilingual individuals (Mechelli et al., 2004). Here, the number of years of yoga experience was positively correlated with GM volume in clusters located in the left mid-insula, left frontal operculum, right posterior middle temporal cortex, and left OFC suggesting that persevering on the yoga path continues to bring positive changes to the brain even in experienced practitioners (minimum experience here was six years). GM volumes or cortical thickness within the insula, temporal cortex, and OFC, albeit sometimes at different locations, have been reported to correlate with both yoga and meditation experience (Lazar et al., 2005; Holzel et al., 2008; Vestergaard-Poulsen et al., 2009; Froeliger et al., 2012; Fox et al., 2014).

### Experience-Related Findings Were Mostly Lateralized to the Left Hemisphere

In the current study, most brain regions’ volume correlating with the number of years of yoga experience was located in the left hemisphere. This was also the case in another study of hatha yoga practitioners with strikingly similar age, education, and yoga experience (Froeliger et al., 2012). According to the homeostasis model of awareness, the left forebrain is associated with energy nourishment, parasympathetic activity, relaxation, approach behaviors, and group-oriented (affiliative) emotions, while the right forebrain is associated with energy expenditure, sympathetic activity, arousal withdrawal (aversive) behavior and individual- oriented (survival) emotions (Craig, 2009). This model fits with an accumulating body of psychophysical literature finding that the left and right forebrains are associated with positive and negative affect, respectively, and with findings related to meditation on joy and happiness, two positive states, reported to almost exclusively activate brain regions in the left hemisphere (Lou et al., 1999). This suggests that increasing years of yoga practice progressively tunes the brain toward a parasympathetically driven mode and positive affective states.

### Experience-Related Findings in the Mid-Insula

The mid-insula is implicated in autonomic integration (Craig, 2011) suggesting that control of internal states may take a while to develop and continue to develop over years, for example through yoga experience. In support of this interpretation, an 8-week intervention including meditation and yoga, increased hippocampal GM volume but did not change insular GM, a region of interest in that study (Holzel et al., 2011). Perhaps related to our finding of increased left mid-insular GM volume as a function of yoga experience, is the finding of Lutz et al. (2013) and colleagues showing a reduced baseline activation in the left anterior insula during pain anticipation that correlated with lifetime meditation experience. Given that posterior-to-anterior processing in the insula is thought to mediate subjective sensations via homeostatic sensory integration (Craig, 2011), and given our finding that this group of yogis tolerated pain significantly longer than matched controls (Villemure et al., 2013), it seems reasonable to speculate that yogis could also show a reduced baseline left anterior insular activation during pain anticipation correlating with experience, perhaps mediated by increased insular volume found in the current study.

### Experience-Related Findings in the OFC

Larger OFC GM volume could be related to better emotional regulation with increasing yoga experience (Kringelbach, 2005). OFC activation was involved in the reduction of pain unpleasantness ratings when novice mindfulness practitioners meditated while exposed to noxious stimulation (Zeidan et al., 2011) suggesting that this area is involved in emotion regulation stemming from a meditative practice. It is therefore possible that increasing yoga experience, which is a meditative practice, could result in increased OFC GM volume over time.

### Experience-Related Findings in the Frontal Operculum

It is unclear why larger left frontal operculum volumes (BA 44) were found in the most experienced yogis since traditionally the function of BA 44, a part of Broca’s area, is ascribed to language (see Friederici, 2011 for a review). However, Broca’s area is also involved in non-language processing domains and has been suggested to generally support hierarchical sequence processing (Friederici, 2011). As such, increasing experience in a non-linguistic domain involving sequence processing (music) was previously reported to increase left Broca’s area GM density (Sluming et al., 2002). Yoga practice requires sequencing movements and breath control exercises in a hierarchical fashion to create a practice where each element leads to the next, promoting a smooth transition toward an ultimate goal. Larger left BA 44 in the most experienced yogis might be related to this process since more experienced practitioners are more likely to rely on their own sequencing in their personal practice than on that of a third party, such as a teacher. In fact, most yoga practitioners in our study were also yoga teachers and the more experienced yogis devoted more time teaching yoga each week. It is therefore reasonable to presume that they were also more proficient in designing yoga sequences.

### Experience-Related Findings in Middle Temporal Gyrus

Finally, more yoga experience was associated with larger GM volume in the right middle temporal gyrus (BA 21). A somewhat comparable region, although in the left hemisphere (peak *x, y, z* coordinates: –51, –31, –9; ours: 64, –31, –6), was found to correlate with meditation experience (Leung et al., 2013). A similar region (peak *x, y, z* coordinates: 54, –32, –7) was among a network of brain regions reported to monitor the transition from innocuous to painful sensation (Johnstone et al., 2012). Yoga practitioners usually closely monitor this transition in order to increase flexibility but avoid stretching to levels that would induce pain, which may signal potential damage to the body. It is, however, unclear why this neuroanatomical change would take years to occur. Alternatively, the temporal lobe has also been implicated in mystical experiences characterized by insights into the unity of all reality and a feeling of positive affect of peace and joy (Saver and Rabin, 1997). For example, a similar area within the right temporal lobe (BA 21) was one of the brain areas activated when Carmelite nuns reported being in a state of union with God (Beauregard and Paquette, 2006). We do not know whether the yogis tested in the present study had ever experienced similar insights deriving from their yoga practice. Therefore, such an explanation remains highly speculative. However, the ultimate goal of yoga as described in the *Yoga Sutras* of Patanjali is to experience such union or oneness – referred to as *Samadhi* in Sanskrit. In any case, acquiring insights into the unity of all reality is likely to require years of practice.

### GM Volume Related to Weekly Practice

Short-term activity-dependent plasticity in the adult human brain is a known phenomenon and can occur very rapidly. These activity-dependent GM changes are accompanied by perceptual and/or performance changes, and usually regress shortly after the activity is terminated. For example, it was previously demonstrated that short-term but repeated presentation of painful stimuli over 8 days increases both pain thresholds and GM density in S1 cortex and that these changes recede after regular nociceptive input is discontinued (Teutsch et al., 2008). Similarly, short-term training for a specific visuo-motor skill results in transient spatially selective increase in GM volume reverting to original size shortly after cessation of training and regression of skill performance (Draganski et al., 2004). This suggests that even if certain brain areas can rapidly show volume changes in the face of exposure or training, continuous training might be necessary to maintain skill/performance/benefits and GM volume increase. This could imply that less practice would not sustain GM volume gains as well as more practice and is likely related to our findings that more time devoted to yoga practice weekly impacts some areas of the brain recruited during the practice, likely helping in the further development and maintenance of structural changes even in experienced practitioners. Indeed, we found evidence of practice-dependent brain alterations related to the number of hours devoted to yoga each week in the right S1/SPL, left hippocampus, midline precuneus/PCC, and right V1.

### S1/SPL and Precuneus/PCC Volumetric GM Alterations Associated with Weekly Yoga Practice

Yoga involves interoceptive awareness and focused attention. During the practice of yoga postures, attention is consciously directed to the breath, body alignment and position, and emotional state. This might be related to alterations in S1 cortex, which contains a representational map of the entire body receiving increasing sensory input with increasing hours of weekly practice, as well as alterations in SPL, involved in the voluntary orienting of attention (Corbetta and Shulman, 2002), and precuneus/PCC, which belong to a network of midline structures thought to be crucial for the integration of self-referential stimuli in the emotional and autobiographical context (Northoff and Bermpohl, 2004; Holzel et al., 2011). Increasing gray matter (GM) volume in all these areas could reflect enhanced somatic awareness related to an increasing weekly amount of yoga practice. Rank ordering of variable importance using the multiple regression product measures, revealed that the practice of postures contributed more to the prediction of S1/SPL volume, while yoga-related meditation contributed more to the prediction of the volume of precuneus/PCC. This is in accordance with the nature of the processing taking place in those structures.

### V1 GM Alterations Associated with Weekly Yoga Practice

Larger GM volume in V1 with increasing weekly practice may be related to some yogic practices involving visualization of objects or scenery, such as some meditation/relaxation techniques including *yoga nidra*, and some breath control exercises requiring visualizing the passage of air through the respiratory system or visualizing that the breath reaches different parts of the body. Indeed, V1 can be activated in neuroimaging studies of visual mental imagery (Kosslyn and Thompson, 2003). However, it must be noted that we did not specifically document the use of visualization practices in the current sample of subjects. It is nevertheless worth noting that V1 was the only brain structure whose volume correlated with the numbers of hours of weekly breath control practice. Furthermore, The beta coefficient related to breath control exercises was significant only for V1 and associated with a large structure coefficient indicating the number of hours of breath control exercises was a good predictor of V1 volume. The commonality analysis showed that the combination of meditation and breath control exercises accounted for almost half the variance in V1.

### Hippocampal GM Alterations Associated with Weekly Yoga Practice

Spending more time doing yoga each week was associated with larger left hippocampal GM volume. Previous studies found that both hippocampi were activated in yoga teachers during *yoga nidra*, a relaxation/meditation technique (Lou et al., 1999) while the left hippocampus was activated during a Kundalini yoga meditation involving attending to the breath while silently repeating Sanskrit words (Lazar et al., 2000). Increased left hippocampal GM was also recently reported following an 8-week mindfulness-based stress reduction intervention including sitting meditation and yoga (Holzel et al., 2011) while significantly greater hippocampal GM volume/density have been repeatedly found in experienced meditators than controls either in the right (Holzel et al., 2008; Luders et al., 2009) or left hippocampus (Luders et al., 2013a,b) with hippocampal volume correlating with experience (Luders et al., 2013a). In older adults, greater aerobic fitness and cardiopulmonary function were also associated with increased hippocampal volume (Pereira et al., 2007; Erickson et al., 2009). Here, the product measure derived from the multiple regression analysis indicated that both the number of hours devoted to yoga postures and to yoga-related meditation were almost equally contributing to the prediction of the left hippocampal volume while the commonality analysis showed that nearly half of the explained variance was related to the combination of postures and meditation. A recent literature review has linked the hippocampus and the regulation of stress hormones (Goosens, 2011) and yoga has been shown to decrease stress and/or anxiety symptoms [for a review see Li and Goldsmith, 2012]. Importantly, a relationship between hippocampal volume and cortisol response to stressors has been reported in humans, with larger hippocampi associated with lower cortisol secretion in response to stressors (Pruessner et al., 2005). Additionally, Luders et al. (2013a), using refined cytoarchitectonic probabilistic maps of peri-hippocampal subsections, found that the effect of meditation on hippocampal volume were specific to the subiculum, a structure known to play a key role in stress regulation (Luders et al., 2013a).

Together these findings suggest that more is better when it comes to the frequency of yoga practice notably as far as somatosensation, attention, self-relevant processing, and stress regulation are concerned. This dose-response should be taken into account in eventual longitudinal studies evaluating the effects of yoga practice on regional brain volume changes.

### Limitations

Our relatively small sample size may have prevented us from identifying subtler GM differences. Matching groups on the amount of exercise performed outside yoga may be viewed as a limitation given that postures may be considered a form of physical exercise and, as mentioned before, physical activity and cardiovascular function can impact brain structure. The global GM differences observed between groups could hypothetically be entirely attributed to this extra amount of physical activity. However, postures are an integral part of yoga practice and are more than a simple physical activity, as practitioners are trained to do them with mindful awareness. Further, it can be argued that a relatively small proportion of *asana*, notably “sun salutations,” involve aerobic exercise that may improve cardiovascular function if a sufficient number of series are performed vigorously enough (i.e., at age-predicted 80% heart rate max; Mody, 2011). Finally, within our yoga group, only a proportion of the effects of weekly yoga practice on the brain were attributable to *asana* and this proportion varied depending on the brain region, with some regions being only modestly influenced by postures.

The use of a higher-resolution head coil (such as the recently developed 32-channel coil) might have permitted us to detect more subtle GM differences, though recent research (Streitburger et al., 2014) suggests that the MP-RAGE sequence used in the current study does not benefit significantly from the potential advantages of a 32-channel coil. Another limitation is that VBM does not allow us to determine what underlies the observed GM changes described here. GM changes could result from many causes, one likely explanation being synaptic changes such as increased spine density associated with synapse formation (Trachtenberg et al., 2002) and pre-synaptic neuronal remodeling (Lerch et al., 2011). This last study directly evaluated the mechanism of experience-dependent volumetric changes in a rodent model of maze learning and found that changes in VBM correlated with Gap-34 staining, a marker of neuronal process remodeling (Lerch et al., 2011). Although this study did not measure changes in dendritic spines, the axonal/presynaptic changes found are thought to occur in parallel with the formation and persistence of specific postsynaptic dendritic spines and associated synapses (reviewed in Fu and Zuo, 2011). Additionally, there is evidence to suggest that glial cell alterations also support structural neuroplasticity (Blumenfeld-Katzir et al., 2011; Johansen-Berg et al., 2012; Sagi et al., 2012).

Despite the cross-sectional nature of this study, the current findings (the correlations between GM volume of several brain areas and yoga experience and practice frequency, the relationship between different aspects of yoga practice and changes in different brain areas, and the possible evidence against age-related whole-brain GM decline in yogis) suggest that yoga practice contributes to the observed brain differences. However, we cannot totally exclude that our sample of yogis had fundamentally different brains to begin with, predisposing them to adopt yoga practice and/or persevering on that path. In fact, some of our results suggest that yoga practitioners might have started with smaller brain volumes in certain brain areas before reaching higher levels of experience. This was particularly evident for the brain areas correlating with the number of years of experience. For example, **Figure 3A** shows that increasing yoga experience is associated with increasing left OFC volume. It also shows that the average OFC volume of the matched control subjects was that of a yoga practitioner with approximately 10 years of experience. As previously mentioned, OFC is involved in emotional regulation and the left hemisphere is mostly related to positive affect. This opens the intriguing possibility that individuals with smaller left OFC might be drawn to yoga practice and, as their left OFC volume increases with yoga practice, and presumably as the benefits of having a larger OFC volume manifest, they continue to persevere on the yoga path. Interestingly, we reported in our previous manuscript that one of the most cited reasons to do yoga in this particular group of subjects was to improve mood (Villemure et al., 2013). However, this remains speculative and longitudinal studies are needed to determine how much of these observations are related to predisposing factors and how much are related to actual yoga practice.

## Conclusion

In conclusion, regular practice of yoga may have neuroprotective effects against whole brain age-related GM decline. Additionally, our results suggest that more weekly regular yoga practice is associated with larger brain volume in areas involved in bodily representation, attention, self-relevant processing, visualization, and stress regulation. Distinct components of yoga practice (postures, breathing exercises, and meditation) or combination of these predicted GM volumes of these brain areas differently, in keeping with the nature of the processing taking place in those structures. Furthermore, certain brain changes continue to occur after several years of practice, as reflected by the link between increasing yoga experience and increasing brain volume in areas subserving autonomic integration, emotional processing and regulation, hierarchical sequential organization, and in a brain area implicated in either the monitoring of the transition between innocuous to painful sensation or in experiences characterized by insights into the unity of all reality and feelings of peace and joy. Most of these experience-related changes were located in the left hemisphere suggesting that increasing years of yoga practice progressively tunes the brain toward a parasympathetically driven mode and positive affective states. Together these findings provide a neural basis for some of the beneficial effects of yoga. Finally, the current study involved yoga practitioners who were otherwise typical North Americans. As such, if the observed structural brain variances are indeed related to yoga training, they should be within the reach of the average person and not reserved to a select few.

## Conflict of Interest Statement

The authors declare that the research was conducted in the absence of any commercial or financial relationships that could be construed as a potential conflict of interest.

## Footnotes

1 http://dbm.neuro.uni-jena.de/vbm/

2 http://www.fil.ion.ucl.ac.uk/spm/
